# Intrinsic cardiomyopathy in pediatric Marfan syndrome: predictive factors and risk assessments

**DOI:** 10.1038/s41390-024-03613-6

**Published:** 2024-10-08

**Authors:** Jonas Kneußel, Jakob Olfe, Veronika C. Stark, Yskert von Kodolitsch, Rainer G. Kozlik-Feldmann, Ingeborg Friehs, Kerstin Kutsche, Michael Hübler, Thomas S. Mir, Daniel Diaz-Gil

**Affiliations:** 1 Department of Pediatric Heart Medicine and Adults with Congenital Heart Disease, University Heart and Vascular Center Hamburg, University Medical Center Hamburg-Eppendorf, Hamburg, Germany; 2 Department of Cardiology, University Heart and Vascular Center, University Medical Center Hamburg-Eppendorf, Hamburg, Germany; 3 Department of Cardiac Surgery, Boston, Children’s Hospital, Boston, MA USA; 4 Department of Surgery, Harvard Medical School, Boston, MA USA; 5 Institute of Human Genetics, University Medical Center Hamburg-Eppendorf, Hamburg, Germany; 6 Department of Pediatrics, Boston Children’s Hospital, Boston, MA USA; 7 Department of Pediatrics, Harvard Medical School, Boston, MA USA; 8 Department of Pediatrics, Boston University Chobanian & Avedisian School of Medicine, Boston, MA USA

## Abstract

**Background:**

Marfan syndrome (MFS) is associated with cardiovascular complications, particularly valvulopathies; however, its association with primary cardiomyopathy remains unclear.

**Methods:**

This retrospective cohort study examined the cardiomyopathy characteristics (CMCs) in pediatric patients with MFS. CMCs were defined as meeting at least one of the following echocardiography or clinical parameters: (1) cardiac index (CI) too low for patient’s age, (2) ejection fraction (EF) <50%, and (3) diastolic dysfunction. The predictive factors for CMCs were determined using a multivariable logistic regression model.

**Results:**

Among 83 patients with MFS (age, median [range], 12.5 [0.4–22.3] years), 39.8% exhibited CMCs. Only 4 patients (5%) showed heart failure symptoms (NYHA > 1). Independent predictors for CMCs included a systemic score of ≥7 (revised Ghent criteria) and likely pathogenic or pathogenic variants in FBN1, including variants that introduce a premature stop codon, splice site variants, and missense variants involving cysteine. A multivariable score was constructed with an AUC of 0.733.

**Conclusion:**

This study offers valuable insights into the prevalence and predictors of CMC in pediatric patients with MFS and presents potential strategies for risk assessment of cardiomyopathy.

**Impact:**

- The objective of this study was to elucidate the contentious issue of intrinsic cardiomyopathy in Marfan syndrome and demonstrate its notable occurrence even in pediatric patients who do not exhibit heart failure symptoms or valvular complications.
- We highlighted the importance of specific FBN1 variants and higher systemic scores in identifying the potential for intrinsic cardiomyopathy in pediatric patients with Marfan syndrome.

## Introduction

Marfan syndrome (MFS) is an inherited connective tissue disorder caused by heterozygous variants of the *FBN1* gene, which encodes fibrillin-1.^1^ This autosomal-dominant condition affects both sexes equally and is estimated to occur in 1 out of every 5000–10,000 live births.^2^ Central to the pathophysiology of MFS is the genetic variant in the *FBN1* gene, which leads to abnormalities in the extracellular matrix microfibrils and disrupts the integrity of connective tissues.^3–5^ The diverse manifestations of the syndrome in various organ systems have made it a subject of extensive study, with cardiovascular complications being the most significant concern.^6^

One of the most notable features of MFS is the dilation of the aorta at the level of the sinuses of the Valsalva. The extent of these aortic abnormalities can vary, with some patients showing aortic root dilation as early as five years old, and more than half of patients experiencing it before the age of 19 years.^6^ In addition to vascular abnormalities, valvular involvement is also common in MFS, with both mitral and tricuspid valve prolapse frequently observed and valvular regurgitation, particularly of the aortic and mitral valves, contributing to the development of heart failure.^3,7,8^

There is growing evidence of primary intrinsic cardiomyopathy in MFS, which is separate from valvular disease and affects both the left and right ventricles.^8–11^ However, this condition is rarely accompanied by clinical symptoms. Research using imaging techniques, such as magnetic resonance imaging (MRI) and echocardiography, has helped understand the cardiac changes in MFS, but our knowledge of the extent of Marfan cardiomyopathy in humans is limited despite extensive research in animal models.^12–15^

Understanding the potential associated cardiac complications in patients with MFS is vital for its effective management. Although the clinical and genetic aspects of MFS have been well studied, there is a notable gap in understanding the risk factors for Marfan cardiomyopathy. This is crucial because evidence suggests that heart failure, in which intrinsic cardiomyopathy is a critical component, is a major MFS complication later in life.^8,16–18^ The estimated prevalence of cardiomyopathic characteristics in pediatric patients with MFS ranges from 3 to 68%,^17^ and a recent meta-analysis revealed that pediatric patients with MFS generally demonstrate larger left ventricular volumes and diminished left ventricular function compared to control groups.^19^ These findings underscore the importance of timely recognition of intrinsic cardiomyopathy in pediatric MFS patients, as early intervention and proper monitoring can be instrumental in averting potential complications and improving the quality of life of these individuals.

To bridge this knowledge gap and gain insights into MFS-related intrinsic cardiomyopathy, we conducted a retrospective observational study involving 83 pediatric patients with MFS. Our goal was to identify the predictors of Marfan cardiomyopathy and establish a scoring system to quantify its occurrence in this patient group. We hypothesized that specific genetic variants combined with distinct phenotypic features may be associated with an increased risk of developing Marfan cardiomyopathy.

## Methods

**Fig. 1:**
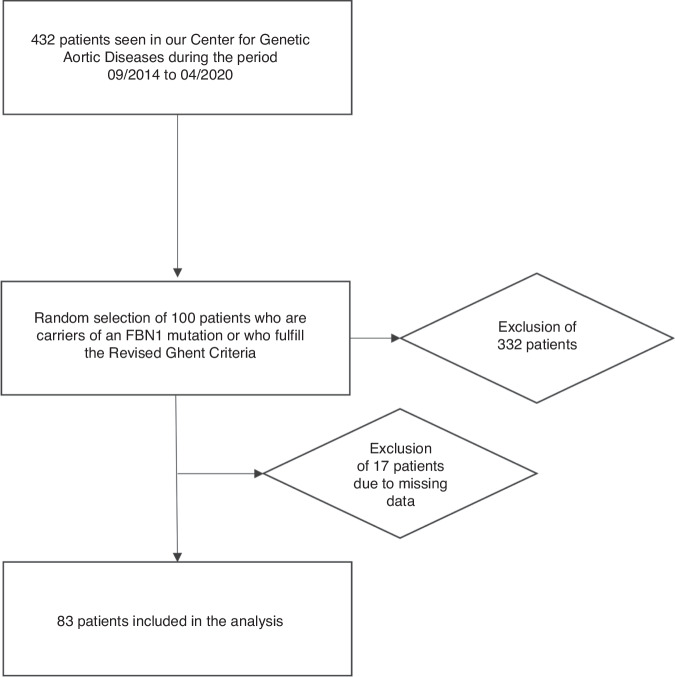
Patient inclusion flowchart. Of the 432 patients seen in our clinic during the observational period, 100 with Marfan syndrome were randomly selected for inclusion in the study. Of these, 83 patients with complete data were included in the final analyses.

Our study included children and adolescent patients under 26 years of age with a confirmed diagnosis of Marfan syndrome (MFS) in the Department of Pediatric Cardiology at the University Heart and Vascular Centre UKE Hamburg. We used the clinical database of our specialized pediatric Marfan clinic, as approved by the Ethics Committee of the Hamburg Medical Association under number 2020-10341-BO-ff. All patients who visited our clinic between 2014 and 2020 were considered for inclusion in the study. To be included, patients had to have Marfan syndrome confirmed by the Revised Ghent Criteria^1^ or a likely pathogenic or pathogenic variant (ACMG Classification) in the FBN1 gene found on genetic testing at the time of inclusion.^20^ The stepwise exclusion process is illustrated in Fig. 1.

In our single-center observational cohort study, we evaluated the cardiomyopathic characteristics of children and adolescents with MFS using predetermined criteria for Marfan cardiomyopathy, including the following:

- Echocardiographically determined cardiac index below the age-adjusted normal limit.^21^ This was calculated using the stroke volume from end-systolic and end-diastolic volume estimations on fractional shortening (FS) measurements. Stroke volumes were multiplied by the heart rate from ECG tracings at the time of FS measurement and divided by the patients’ body surface area (DuBois formula).
- Reduced ejection fraction less than 50% based on fractional shortening in echocardiography, or
- echocardiographic signs of diastolic dysfunction (abnormal E/A in mitral valve Doppler inflow or E/E’ in mitral valve tissue Doppler for age).^22^

To rule out inaccuracies due to asynchrony of cardiac contraction, we examined the ECG findings of all patients at the time of consultation for the occurrence of bundle branch blocks. Based on the above criteria, we generated a composite primary outcome variable, termed “cardiomyopathic characteristics” (CMC). This composite outcome served as the basis for univariable and multivariable logistic regression analyses. We selected this composite outcome, which includes both systolic and diastolic dysfunction, to increase the statistical power and the probability of discovery in the study, given the broad spectrum of cardiomyopathic manifestations in Marfan syndrome. Clinical symptoms at the time of inclusion were assessed using the NYHA classification in patients older than 6 years (NYHA 2–4) and the Ross classification in patients 6 years old or younger (Ross classes 2–4) at the time of evaluation.^23^

### Genetic variants

**Fig. 2:**
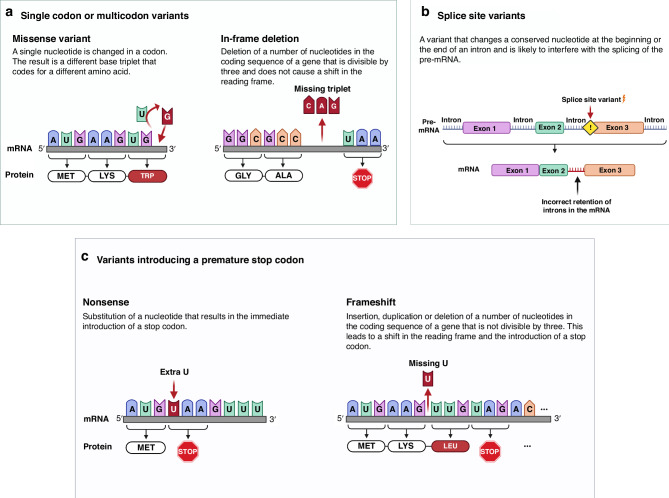
Schematic illustration of variant classification used in this study. **a** Single codon or multicodon variants including missense variants and in-frame deletions. **b** Splice site variants. **c** Variants introducing a premature stop codon including nonsense and frameshift variants. Created using BioRender.com. Adapted from “Frameshift Mutations” and “RNA Processing in Eukaryotes 2” by BioRender.com (2023).

We employed the classification method previously described by our group,^24^ specifically to conduct a comparative effect analysis on our primary outcome (Fig. 2).

- Substitutions and in-frame deletions: This group included variants that affect one or more codons, such as missense variants and in-frame deletions that leave the reading frame intact. Missense variants were further subcategorized based on whether they replace or generate a cysteine residue (missense variants involving cysteine) or result in another amino acid substitution (missense variants w/o cysteine involvement).
- Splice site variants.
- Variants introducing a premature stop codon: This group consists of nonsense and frameshift variants.

### Statistical analysis

Echocardiographic, phenotypic, and genotypic predictors of impaired heart function were evaluated as dependent variables, and the binary composite outcome variable, CMC, was evaluated as an independent variable. Hypothesis-driven regression models were constructed based on all identified potential confounders using a priori clinical and pathophysiological knowledge. Descriptive analytics and visual inspection techniques, including histograms, density plots, and Q–Q plots, were used to assess the normality of the distribution. The Shapiro–Wilk test was used to confirm normality, with a *P*-value greater than 0.05 indicating a normal distribution. Continuous variables with a normal distribution were reported as mean ± standard deviation, ordinal variables as median (range), and categorical variables as percentages (frequency) unless otherwise specified. For data that were missing at random, filter methods were employed to reduce potential biases during statistical analysis. This involved computing critical statistical measures solely from the available dataset and excluding missing values.

A multivariate model was fitted using the relevant variables. For all continuous variables in the model, the receiver operating characteristic (ROC) curve was used to determine the optimal cutoff points using Youden’s J-Index, which maximized the combination of sensitivity and specificity. A point value was assigned to each final predictor level proportional to the odds ratio from the binary logistic regression model (rounded to full numbers) to create a prognostic score for Marfan cardiomyopathy. To evaluate the predictive accuracy of the score, we conducted an area under-the-curve (AUC) analysis using the receiver operating characteristic (ROC) curve. The score was validated internally using 2000 bootstrap resamples. The validation statistics assessed included the AUC to assess discrimination, and the bias-corrected Somers’ D rank correlation, Nagelkerke’s R-squared, and Brier quadratic probability score to assess calibration.^25^ Statistical analysis was performed using STATA 16.1 (StataCorp LLC, College Station, TX). All reported *P*-values were 2-sided with a minimum significance of 0.05.

## Results

**Fig. 3:**
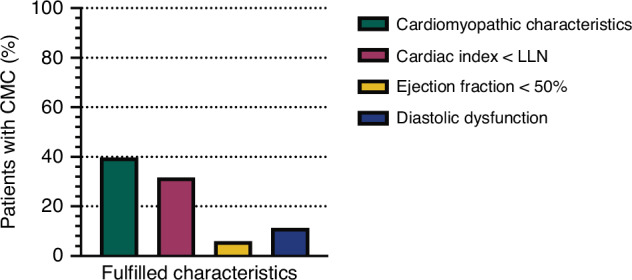
Cardiomyopathic characteristics of the study population. In total, 39.8% of patients (*n* = 33) had cardiomyopathic characteristics (green). Most of these patients (31.7% of included patients) had a cardiac index below the normal range for the patient’s age (*n* = 26; pink). An ejection fraction below 50% was found in 6.0% of patients (*n* = 5; yellow). Diastolic dysfunction was present in 11.3% (*n* = 8; blue) of patients. LLN lower limit of normal. CMC cardiomyopathic characteristics.

A total of 83 children and adolescents with Marfan syndrome were included in this study, and their characteristics are summarized in Table 1. The median age of the participants was 12.5 years, ranging from 0.4 to 22.3 years. Of these, 33 (39.8%) satisfied the predefined criteria for Marfan cardiomyopathy (Fig. 3). The likely pathogenic or pathogenic FBN1 variants identified in our study subjects are summarized in the supplementary materials (Table S1). Notably, 78.8% of these patients (*n* = 26) had a cardiac index below the age-adjusted lower limit of normal.^21^ In our cohort, 19 patients exhibited an incomplete right bundle branch block, and one patient additionally demonstrated a left anterior hemiblock at the time of CI evaluation. The presence of these conduction abnormalities did not significantly affect our primary outcome. Similarly, the incidence of aortic and mitral regurgitation did not significantly alter the primary outcome variable of the study. A reduced ejection fraction of less than 50% was observed in 6.0% (*n* = 5) of the cohort, and 11.3% (*n* = 8) showed signs of diastolic dysfunction (Fig. 3). No significant association between mitral valve pathology and the binary outcome variable, diastolic dysfunction, was identified. Only 4.8% (*n* = 4) of patients had symptoms of heart failure according to the NYHA classification, and all patients under 6 years old had no heart failure symptoms based on the Ross classification. Our analysis identified four factors that independently predicted the presence of cardiomyopathic characteristics: a systemic score of ≥7 based on RGC, a variant in FBN1 introducing a premature stop codon, a splice site variant, and a missense variant involving cysteine.

**Table 1:** Patient characteristics compared between patients with and without characteristics of Marfan cardiomyopathy.

Variables	Patients without cardiomyopathic characteristics (*n* = 50)	Patients with cardiomyopathic characteristics (*n* = 33)
Age at consultation – years, median (range)	12.7 (0.4–22.3)	11.7 (3.1–21.9)
Sex, *n* (%)	Female 22 (44.0%)	Female 16 (48.5%)
	Male 28 (56.0%)	Male 17 (51.5%)
Cardiovascular features		
Sinus Valsalva dilatation, defined as Z > 2 (%), *n* (%)	21 (42.0%)	9 (27.3%)
Mitral valve prolapses, *n* (%)	25 (51.0%)	16 (51.6%)
Tricuspid valve prolapses, *n* (%)	26 (53.1%)	21 (67.7%)
Pulmonary artery dilatation, *n* (%)	2 (15.4%)	0 (0%)
Aortic valve regurgitation, *n* (%)	None: 41 (82.0%)	None: 29 (87.9%)
	Mild: 8 (16.0%)	Mild: 4 (12.1%)
	Moderate: 1 (2.0%)	Moderate: 0 (0%)
	Severe: 0 (0%)	Severe: 0 (0%)
Mitral valve regurgitation, *n* (%)	None: 19 (38.0%)	None: 17 (51.5%)
	Mild: 22 (44.0%)	Mild: 13 (39.4%)
	Moderate: 9 (18.0%)	Moderate: 2 (6.1%)
	Severe: 0 (0%)	Severe: 1 (3.0%)
Bundle branch block present on ECG, *n* (%)	11 (22.4%)	8 (24.2%)
Surgical aortic valve replacement before study inclusion, *n* (%)	0 (0%)	2 (6.1%)
Skeletal features		
Dural ectasia, *n* (%)	9 (45.0%)	7 (63.6%)
High-arched palate, *n* (%)	15 (60.0%)	18 (81.8%)
Fulfills ≥3 of 5 facial features defined by the Revised Ghent Criteria, *n* (%)	14 (36.8%)	15 (57.7%)
Arm-span-to-height ratio >1.05, *n* (%)	12 (34.3%)	7 (33.3%)
Pectus excavatum, *n* (%)	9 (23.1%)	10 (35.7%)
Pectus carinatum, *n* (%)	6 (15.0%)	2 (8.3%)
Scoliosis, *n* (%)	9 (22.5%)	15 (55.6%)
Wrist and thumb sign, *n* (%)	11 (32.4%)	13 (50.0%)
Hindfoot deformity, *n* (%)	19 (55.9%)	15 (60.0%)
Reduced elbow extension, *n* (%)	4 (12.1%)	5 (23.8%)
Other features		
Positive family history, *n* (%)	35 (70.0%)	13 (57.6%)
Ectopia lentis, *n* (%)	17 (34.0%)	8 (24.2%)
Systemic score ≥7, *n* (%)	19 (38.0%)	21 (63.6%)
Skin striae, *n* (%)	10 (24.4%)	8 (33.3%)
Pneumothorax, *n* (%)	3 (6.0%)	2 (6.1%)
Myopia >3dpt, *n* (%)	7 (22.6%)	7 (31.8%)
Diagnosis		
Based on RGC, *n* (%)	45 (90.0%)	27 (81.8%)
Based on RGC - no genetic testing performed, *n* (%)	5 (10.0%)	2 (6.1%)
Based on RGC - negative genetic result or VUS/benign variant, *n* (%)	2 (4.0%)	1 (3.0%)
Based on RGC with incomplete genetic testing data, *n* (%)	10 (20.8%)	2 (6.5%)
Based on confirmed (likely) pathogenic *FBN1* variant, *n* (%)	33 (66.0%)	28 (84.8%)
Genetic data		
In-frame deletions and missense variants without cysteine involvement, *n* (%)	10 (20.0%)	3 (9.1%)
Missense variants involving cysteine, *n* (%)	13 (26.0%)	11 (33.3%)
Splice site variants, *n* (%)	1 (2.0%)	4 (12.1%)
Nonsense and frameshift variants, *n* (%)	9 (18.0%)	9 (27.3%)
Genetic testing performed but incomplete genetic data and/or VUS/benign variant (diagnosis via RGC), *n* (%)	12 (24%)	4 (12.1%)
No genetic testing performed, *n* (%)	5 (10.0%)	2 (6.1%)

*RGC* Revised Ghent Criteria, *VUS* Variant of uncertain significance, *FBN1* Fibrillin-1.

**Fig. 4:**
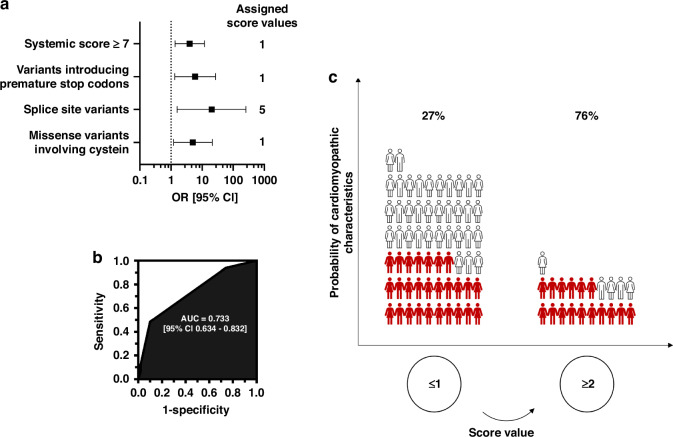
Multivariable prediction of CMCs. **a** Multivariable prediction model with assigned point values proportional to odds ratios. **b** Receiver operating characteristic (ROC) curve for prediction of CMCs recurrence with a score system established using multivariable regression. **c** Empirical comparison of CMCs rates stratified by our prediction model at a cutoff value of two points.

These variables were incorporated into a multivariable risk model; based on the odds ratio, they were assigned point values of 1, 1, 5, and 1 for a systemic score equal to or greater than 7, a genetic variant in FBN1 introducing a premature stop codon, a splice site variant, and a missense variant involving cysteine, respectively (Fig. 4a). The cumulative point values for the prediction scores ranged from 0 to 6 (median 1 [IQR, 1–2], with a maximum point value of 6). The risk of developing cardiomyopathic characteristics increased more than 2-fold with each one-point increase in the score (OR 2.3, 95% CI, 1.2–4.2; *P* = 0.009). Internal validation of the risk score using 2000 bootstrap resamples revealed strong model discrimination with an AUC of 0.733 (95% CI 0.634–0.843, Fig. 4b) and good calibration with a bias-corrected Somers’ D rank correlation of 0.46, Nagelkerke’s R-squared of 0.15, and Brier quadratic probability score of 0.21. This indicates the good internal validity of the scoring system.

A cardiomyopathy score of 1 or less was associated with a 27.4% estimated probability of Marfan cardiomyopathy, whereas a score of 2 points or more indicated a 76.2% probability (Fig. 4c). A score ≥2 had a specificity of 90.0% for CMCs.

The abovementioned predictors were independent of the long-term use of angiotensin 2 type 1 receptor (AT2R1) blockers (>5 years), Sinus of Valsalva diameter Z-scores, and valvular abnormalities, including aortic regurgitation, mitral regurgitation, and mitral valve prolapse.

## Discussion

The primary finding of this study revealed that a significant proportion of children and adolescents with Marfan syndrome (MFS) exhibited signs of Marfan cardiomyopathy, despite not having symptoms related to heart failure. We identified four critical risk factors: a systemic score of 7 or higher, a genetic variant in FBN1 introducing a premature stop codon, a splice site variant, and a missense variant involving cysteine, which helped to identify patients at the highest risk for Marfan cardiomyopathy. By developing a prediction model with an area under the curve (AUC) of 0.73 and strong validity, we aim to assist in assessing the risk of Marfan cardiomyopathy in children and adolescents receiving outpatient treatment.

Genetic variations linked to Marfan syndrome have been shown to affect the severity of the condition.^26^ More than 3000 distinct disease-causing variants have been identified, primarily within affected families.^27^ Our study aligns with previous research^24,28–30^ which found that patients with missense variants involving cysteine residues generally experience a more severe form of the disease. Cysteine is essential for the tertiary structure of the fibrillin-1 protein, with numerous domains comprised of cysteine residues forming disulfide bridges.^31,32^ Any variation at these sites can result in misfolding and subsequent dysfunction of the fibrillin protein, often serving as the genetic basis for more severe Marfan syndrome symptoms.^33^ Our research suggests that pediatric patients with missense variants involving cysteine are more likely to experience CMCs, which is consistent with previous findings of an increased risk for aortic dilation requiring drug treatment, tricuspid valve prolapse, and severe myopia in those with other missense variants.^24^

In addition, we discovered that FBN1 variants that lead to premature stop codons, such as nonsense, frameshift, and pathogenic splice site variants, were associated with an increased likelihood of developing CMCs. These variants most likely cause haploinsufficiency, where a single functional copy of a gene is insufficient to produce the amount of protein necessary for normal cellular function.^31^ These patients tend to experience a more severe clinical course, as indicated by the higher incidence of aortic dissection and cardiovascular-related deaths. The risk of cardiovascular mortality is 2.5 times greater than that of the general population.^31,34,35^ Although the exact role of FBN1 variants in intrinsic cardiomyopathy remains unclear,^17^ individuals with haploinsufficiency-causing variants in FBN1 have been found to have dilated left ventricles and reduced left ventricular function.^36–38^ Supporting these findings, a study by Abd El Rahman et al. highlights the significant impact of severe FBN1 gene mutations on left ventricular function, further elucidating the molecular mechanisms underlying Marfan syndrome and its cardiac manifestations.^39^

Our study results align with those of prior investigations on primary cardiomyopathy in Marfan syndrome.^35,39^ In 2002, Porciani et al. reported subclinical changes in patients with MFS that were independent of valve disease and may indicate early myocardial involvement. These changes include a significant increase in left ventricular mass and a decrease in diastolic function, suggesting potential early indicators of primary myocardial involvement.^40^ Alpendurada et al. conducted a study of 68 adult patients with Marfan syndrome, demonstrating biventricular enlargement and dysfunction, which are usually mild and asymptomatic. Some patients exhibit reduced left ventricular ejection fraction, although this reduction is not associated with aortic regurgitation or other contributing factors.^8^

Additionally, a meta-analysis by Xu et al. reinforced the idea of intrinsic cardiac impairment in Marfan syndrome. Their comprehensive analysis showed that patients with Marfan syndrome generally have larger left ventricular volumes and poorer left ventricular function than control groups, regardless of common risk factors.^19^

Despite the typically asymptomatic nature of the initial cardiac changes in Marfan syndrome, their long-term clinical significance is evident. Heart failure is a significant concern in MFS, with studies indicating that it is one of the primary causes of death, even on par with aortic dissection.^16–18^ While the causes of heart failure in such cases are often attributed to underlying severe valvular disease, there is also evidence of intrinsic myocardial dysfunction in patients with MFS due to the aforementioned mechanisms. This dysfunction is usually mild, clinically unremarkable, and does not progress significantly over time. Nevertheless, some patients may eventually develop fulminant clinical heart failure.^16–18^

The precise mechanisms underlying myocardial dysfunction in patients with MFS remain unclear, although animal experiments have demonstrated that fibrillin-1, which is present in the heart and is integral to the architecture of cardiac muscle tissue,^15^ may play a role. Furthermore, cardiomyopathy can develop independent of aortic or valvular abnormalities, suggesting intrinsic cardiac dysfunction.^17,41^

In conclusion, our study highlights the critical predictors of primary cardiomyopathy in Marfan syndrome, specifically the genetic variants that have a severe impact on the structure or amount of fibrillin-1 and lead to more severe organ involvement. Our findings underscore the importance of closely monitoring high-risk patients. This knowledge can enhance the risk assessment and improve patient care. Moreover, additional research and larger prospective studies are required to further refine the management and prognosis of patients with Marfan cardiomyopathy.

## Limitations

This study has several important limitations that should be considered when interpreting the results. Firstly, its retrospective design introduces potential sources of bias and may limit the completeness of the data because of its reliance on historical records. Moreover, missing data is a common issue in retrospective research, and their presence in this study can impact the overall robustness of the findings.

Secondly, the method used to assess the cardiac index, based on echocardiographic estimations derived from the Teichholz method using fractional shortening measurements and heart rates, has inherent limitations and assumptions that potentially affect the accuracy of cardiac function assessment. Alternative methods, such as using aortic velocity time integral (VTI) to determine cardiac output, offer advantages but can overestimate stroke volume in patients with aortic valve geometry alteration, as it is the case in Marfan patients.^42–44^ Moreover, the use of the Teichholz method, despite its known limitations in precision, was primarily utilized to broadly categorize patients rather than to precisely predict volumetric measurements. This approach aligns with screening for potential abnormalities rather than achieving precise quantification.

Additionally, the scarcity of diastolic function assessments in the pediatric population is a significant constraint to gaining a comprehensive understanding of diastolic dysfunction in this specific population. While the study’s cross-sectional nature provides valuable insights into cardiac function at a single point in time, it does not offer insights into changes over time or causality. To strengthen the reliability and generalizability of these findings, prospective validation studies are warranted.

## Supplementary information

Supplementary Table

## Data Availability

The datasets generated and analyzed during the current study are not publicly available due to privacy and ethical considerations but are available from the corresponding author on reasonable request. This approach is in accordance with Springer Nature’s policy to support transparency and ethical sharing of data.
